# Piperine protects against pyroptosis in myocardial ischaemia/reperfusion injury by regulating the miR‐383/RP105/AKT signalling pathway

**DOI:** 10.1111/jcmm.15953

**Published:** 2020-11-21

**Authors:** Xin Guo, Shan Hu, Ji‐Jun Liu, Ling Huang, Peng Zhong, Zhi‐Xing Fan, Ping Ye, Man‐Hua Chen

**Affiliations:** ^1^ Department of Cardiology The Central Hospital of Wuhan Tongji Medical College Huazhong University of Science and Technology Wuhan China; ^2^ Heart Function Department The Central Hospital of Wuhan Tongji Medical College Huazhong University of Science and Technology Wuhan China; ^3^ Department of Cardiology Renmin Hospital of Wuhan University Wuhan China

**Keywords:** ischaemia/reperfusion, miR‐383, PI3K/AKT, piperine, pyroptosis, RP105

## Abstract

miRNA‐mediated pyroptosis play crucial effects in the development of myocardial ischaemia/reperfusion (I/R) injury (MIRI). Piperine (PIP) possesses multiple pharmacological effects especially in I/R condition. This study focuses on whether PIP protects MIRI from pyroptosis via miR‐383‐dependent pathway. Rat MIRI model was established by 30 minutes of LAD ligation and 4 hours of reperfusion. Myocardial enzymes, histomorphology, structure and function were detected to evaluate MIRI. Recombinant adenoviral vectors for miR‐383 overexpression or miR‐383 silencing or RP105 knockdown were constructed, respectively. Luciferase reporter analysis was used to confirm RP105 as a target of miR‐383. Pyroptosis‐related markers were measured by Western blotting assay. The results showed that I/R provoked myocardial injury, as shown by the increases of LDH/CK releases, infarcted areas and apoptosis as well as worsened function and structure. Pyroptosis‐related mediators including NLRP3, cleaved caspase‐1, cleaved IL‐1β and IL‐18 were also reinforced after MIRI. However, PIP treatment greatly ameliorated MIRI in parallel with pyroptotic repression. In mechanistic studies, MIRI‐caused elevation of miR‐383 and decrease of RP105/PI3K/AKT pathway were reverted by PIP treatment. Luciferase reporter assay confirmed RP105 as a miR‐383 target. miR‐383 knockdown ameliorated but miR‐383 overexpression facilitated pyroptosis and MIRI. Moreover, the anti‐pyroptotic effect from miR‐383 silencing was verified to be relied on the RP105/PI3K/AKT signalling pathway. Additionally, our present study further indicated the miR‐383/RP105/AKT‐dependent approach resulting from PIP administration against pyroptosis in MIRI. Therefore, PIP treatment attenuates MIRI and pyroptosis by regulating miR‐383/RP105/AKT pathway, and it may provide a therapeutic manner for the treatment of MIRI.

## 1 INTRODUCTION

Currently, the most effective way to save dying cardiomyocytes induced by acute myocardial infarction (AMI) is timely revascularization.^1^, ^2^, ^3^, ^4^, ^5^ However, re‐establishing blood to ischaemic area yields additional myocardial damages known as myocardial ischaemia/reperfusion (I/R) injury (MIRI).^1^, ^2^, ^3^ MIRI affects clinical efficacy of revascularization strategies and serves as an important factor in worsening heart structure and function.^1^, ^2^, ^3^, ^6^, ^7^ Although programmed cell death that mainly contains necrosis, apoptosis and autophagy acts as the major pathogenesis in the development of MIRI,^3^, ^8^, ^9^ it is now verified that pyroptosis‐associated necrosis also plays important roles in MIRI.^6^, ^7^, ^10^

Pyroptosis acts as a pro‐inflammatory programmed cell death and was characterized as a NLRP3‐caspase‐1‐dependent response during MIRI.^6^, ^7^, ^10^, ^11^ When NLRP3 is stimulated, the inactive pro‐caspase‐1 is hydrolysed into activated caspase‐1 subsequently.^6^, ^7^, ^11^ Activated caspase‐1 then acted as a crucial enzyme to stimulate specific targets including interleukin‐1β (IL‐1β) and interleukin‐18 (IL‐18), which recruits pro‐inflammatory response in the initiation and progression of MIRI.^6^, ^7^, ^11^, ^12^ Thus, targeting pyroptosis‐related myocardial death may be a therapeutic approach for the treatment of MIRI.^7^, ^10^, ^12^

Piperine (PIP) is a phenolic substance that found in black pepper and long pepper.^13^, ^14^ miRNAs function as highly conserved non‐coding RNAs and repress target mRNA via direct bonding to 3′UTR, thereby implicating in myocardial pyroptosis and I/R injury.^10^, ^15^ Previous studies have reported that PIP exerts pharmacological effects possible via miRNA‐dependent manners.^16^, ^17^ Recently, the effects of PIP on the modulation of pyroptosis and I/R insults have attracted much attention.^18^, ^19^, ^20^, ^21^ However, the exacted effects of PIP via the miRNA‐related ways in MIRI are not clear, and possible pathogenetic mechanism associated with pyroptosis remains unknown. Interestingly, an altered level of miRNA, miR‐383, has been identified in the development of ischaemic condition^22^ and was involved in the progression of pyroptosis.^23^ Nevertheless, it is not clear to the contributions and molecular mechanisms of miR‐383 in association with PIP and pyroptosis in MIRI.

Emerging investigations from our study and others have revealed that radioprotective 105 kd protein (RP105) plays as one of cell‐surface proteins and seems to be able to trigger intercellular pro‐survival PI3K/AKT signalling pathway in ischaemic condition.^24^, ^25^ Additionally, the activation of RP105/PI3K/AKT axis resulting from specific miRNA inhibition was proved to be a crucial molecular mechanism in the protection of myocardial ischaemia.^25^ Compellingly, by utilizing luciferase reporter assay, our study confirmed RP105 as a potential target gene of miR‐383 in a 3′UTR‐dependent manner. Thus, we herein hypothesized that PIP treatment could inhibit miR‐383 and causally result in the activation of RP105/PI3K/AKT axis, thereby protecting against I/R‐induced cardiac injury and myocardial pyroptosis.

## 2 MATERIALS AND METHODS

### 2.1 Chemicals and reagents

Piperine (PIP) specified to be more than 97% pure was purchased from Shanghai Winherb Medical Science Co. (Shanghai, China).^13^ Lactate dehydrogenase (LDH) and creatine kinase (CK) were detected by commercially available ELISA kits (Jiancheng Bioengineering Institute). The following primary antibodies were from Abcam: anti‐p‐PI3K (1:600 dilution, ab182651), anti‐t‐PI3K (1:400, ab191606), anti‐NLRP3 (1:1000, ab263899), anti‐IL‐18 (1:500, ab191860) and anti‐GAPDH (1:1000, ab37168). The following primary antibodies were obtained from Cell Signaling Technology: anti‐p‐AKT (1:800, #9145), anti‐t‐AKT (1:600, #9139), anti‐cleaved caspase‐1(1:600, #89332), anti‐pro‐caspase‐1 (1:1000, #24232), anti‐cleaved IL‐1β (1:500, #63124) and anti‐pro‐IL‐1β (1:500, #12703). Antibody against RP105 (1:600, PAB18126) was obtained from ABNOVA. The BCA protein assay kit was purchased from Pierce. Evans blue and TTC dying were purchased from Beyotime Institute of Biotechnology. LY294002 (a PI3K/AKT inhibitor) were from Sigma‐Aldrich. The miR‐383 mimic and its scrambled oligonucleotides (miR‐NC) were produced by GenePharma Co., Ltd.

### 2.2 Animal care and the construction of MIRI model

Adult male Sprague Dawley (S‐D) rats (about 6‐8 weeks, 220‐250 g) were purchased from Vital River Laboratories and housed at (23 ± 2)°C under a cycle of 12‐h light/12‐h darkness with free access to food and water.^3^ The procedures for experiments and animal care were approved by the Animal Care and Use Committee of The Central Hospital of Wuhan, Tongji Medical College, Huazhong University of Science and Technology University (Approval Number. 20191808), and conformed to the Guide for the Care and Use of Laboratory Animals produced by the National Institutes of Health.

The rat MIRI model was carried out as previously described with some modifications.^3^, ^26^, ^27^ Pentobarbital sodium (40 mg/kg, Sigma) was intraperitoneally injected to anaesthetize animals. After rats were fixed in the supine position, a median incision in the neck was made to expose trachea. The rats were artificially ventilated with a small animal respirator at 70 strokes per minute. Standard body part II‐lead ECG was monitored throughout the operation. Afterwards, a left thoracotomy was performed and left arterial descending (LAD) coronary artery was located. Next, a small curved needle with a 6‐0 silk suture was passed through the myocardium beneath LAD, and a ligation was placed to block the blood flow. The MIRI model was successfully established when the ST segment in lead II was elevated and the regional myocardial surface that became pale was observed. Thirty minutes later, knot was untied to perfuse myocardium. At 4 hours post‐reperfusion, the animals were killed, and the blood and heart specimens were harvested for further analysis.

### 2.3 Viral establishment and transduction into cardiomyocytes in vivo

Recombinant adenoviral vector encoding miR‐383 and its negative control was obtained from GeneChem, Inc.^3^, ^27^, ^28^ In brief, the entire coding region of miR‐383 was cloned into shuttle vector GV135 (Shanghai, GeneChem, Inc.). Then, 293T cells co‐transfection with shuttle vector and a packaging plasmid was carried out by an AdMax Packaging system. By using Cre/loxP enzymes, Ad‐miR‐383 was synthesized. Afterwards, virus were amplified, and routinely titrated to 1.0 × 10^10^ PFU/mL. As similar methods, adenovirus encoding miR‐383 harbouring RNAi sequence (Ad‐anti‐miR‐383) or RP105 short hairpin RNA (AdshRP105) was generated to knockdown miR‐383 or RP105, respectively. As to viral transduction in the in vivo study, 100 μL of adenovirus solutions were intramyocardially injected 48 hours before LAD occlusion, and adenoviral injection should be averagely divided into five sites as previously described.^3^

### 2.4 Animal pre‐treatments and experimental groups

To measure possible effects of PIP in MIRI, animals were randomly assigned into four groups: (a) Sham + vehicle group; (b) Sham + PIP group; (c) I/R + vehicle group; and (d) I/R + PIP group (Supplementary material, Figure S2A). To induce PIP pre‐treatment, animals were received a gavage of PIP at the indicated dosage of 30 mg/kg (diluted in sterile water) for three consecutive days prior to MIRI. Sterile water treatment was performed as the vehicle with the same method. The optimal drug dose of PIP pre‐treatment was determined by the data from the pre‐tests (Supplementary material, Figure S1).

To further measure the underlying mechanisms of anti‐I/R and anti‐pyroptotic effects of PIP, an additional experiment was performed in which Ad‐miR‐383 or AdshRP105 was administered in the absence or presence of PIP pre‐treatment under MIRI (Supplementary material, Figure S2D). Furthermore, to detect the potential roles of miR‐383 on the expression of RP105/PI3K/AKT pathways under MIRI, rats were divided into five groups: sham group; I/R + group; I/R + Ad‐GFP group; I/R + Ad‐miR‐383 group; and I/R + Ad‐anti‐miR‐383 group (Supplementary material, Figure S2B). To confirm that miR‐383 regulated MIRI and cardiac pyroptosis via the PP105/PI3K/AKT‐dependent avenues, a PI3K/AKT inhibitor (LY294002, 0.3 mg/kg) was injected via a caudal vein 30 minutes prior to LAD occlusion.^3^, ^26^ Moreover, AdshRP105 was used to knockdown RP105 level followed by MIRI (Supplementary material, Figure S2C). At the end of experiments, all animals were killed, and blood sample and heart tissue were collected for the subsequent analysis.

### 2.5 Determination of myocardial enzymes

To assay the levels of necrotic cardiomyocytes after MIRI, the blood samples were collected for biological analysis of CK and LDH.^3^, ^26^ Commercially available biochemical kits (Beijing KEMEI DONGYA Biotechnology Ltd.) were used according to the manufacturer's instructions. LDH and CK were calculated by a microplate spectrophotometer (Shimadzu Corporation). The data are presented as U/litre.

### 2.6 ELISA assays for IL‐18, IL‐1β and NLRP3

The levels of IL‐18 and IL‐1β in blood samples and NLRP3 concentration in heart tissue were detected by commercial ELISA kits (Nanjing Jiancheng Bioengineering Institute, Nanjing, China) as the manufacturer's instructions.^7^

### 2.7 Echocardiographic estimation

Echo assay was used to determine left ventricular (LV) function as previously demonstrated.^3^, ^4^, ^6^ The transthoracic echocardiograms were recorded using a MyLab 30CV ultrasound system (Biosound Esaote, Inc.). As described previously,^3^, ^26^ two‐dimensional (2D) images were collected along parasternal short axes at the level of mid‐papillary muscle for at least three cardiac cycles. LV M‐mode tracing at the mid‐papillary level was measured and averaged to determine the LV ejection fraction (LVEF) and factional shortening (LVFS).

### 2.8 Detection of myocardial infarct area

Evans Blue and TTC staining was performed to evaluate infarcted areas in MIRI as reported previously.^3^, ^4^, ^6^ In brief, LAD was ligated for 30 minutes followed by 4 hours of reperfusion, and then, 2% Evans blue was intravenously injected via jugular veins. Next, rat hearts were harvested, washed in PBS and serially cut into 4 sections at 2 mm thick from the apex to base. After incubation with 2% TTC for 20 minutes, the sections were delineated into three regions including non‐ischaemia (blue), areas at risk (AAR, deep red) and infarcted areas (IA, pale white). The heart sections at papillary muscle plane were captured with a microscope camera and were quantified using Image‐Pro Plus 6.0 (Media Cybernetics). The percentage of infarcted areas was calculated with following formula: IA/total left ventricles (LV) ×100%.

### 2.9 Histological and immunohistochemical examinations

At the end of experiments, the samples of the left ventricle from each group were obtained and immersed in a 4% paraformaldehyde solution for 24 hours. Then, the specimens were embedded in paraffin after fixation was completed, and the samples were cut into 5‐μm‐thick sections on glass slides. Afterwards, haematoxylin and eosin (H&E) staining was performed, and light microscopy was used to observe heart morphology as previously described.^26^, ^29^ To detect the severity of MIRI, five fields of each section were randomly selected. It was then scored by two individuals blinded to all experiments as previously demonstrated in line with criteria^26^, ^29^:0, no damage; 1, mild injury (interstitial oedema and focal necrosis); 2, moderate injury (cardiomyocyte swelling and necrosis); 3, severe injury (formation of necrotic contraction bands and inflammatory cell enrichments); and 4, highly severe injury (expanded necrosis of contraction bands, inflammatory cell infiltration and haemorrhage).

Moreover, the protein expression of NLRP3 was viewed by immunohistochemistry as described in prior study.^29^, ^30^ In detail, after the sections were deparaffinized and hydrated, the hydrogen dioxide was used to block endogenous peroxidase. Slides were then incubated with a primary antibody against NLRP3 at 4°C overnight and were washed by PBS for three times. Positive staining (brown) was visualized via a DAB kit. Haematoxylin was made to re‐stain nucleus. Average optical density (AOD = IOD/Area) was calculated to statistical analysis as previously demonstrated.^29^

### 2.10 Mitochondrial morphology

Ventricular tissues were collected to view mitochondrial structure by transmission electron microscopy (TEM) as previously described.^29^ Dissected heart tissues (1‐2 mm‐wide blocks) were immersed in 4% glutaraldehyde overnight. After samples were post‐fixed in 1% osmium tetroxide for 1 hour, the sections were dehydrated in a graded ethanol series up to 100% and embedded in epoxy resin. Ultrathin sections (80 nm thick) were observed with a JEM‐1400 transmission electron microscope (TEM) (Jeol).

### 2.11 Apoptosis evaluation by TUNEL

Myocardial apoptosis induced by I/R injury was determined by TUNEL staining.^3^, ^26^ Briefly, paraffin‐embedded heart samples were cut into 5‐μm sections. After deparaffinization and rehydration, TUNEL staining was performed as the manufacturer's instruction. Haematoxylin staining was utilized to identify cardiomyocyte nuclei. TUNEL‐positive nuclei were counted from five randomly selected fields in each slice. The nuclei of the apoptotic cells and total cells were stained brown and blue, respectively. The apoptotic rate was calculated as the ratio of TUNEL‐positive cells vs total cardiomyocytes.

### 2.12 Immunofluorescence staining

The expression of RP105 in rat heart was measured by immunofluorescence (IF) and was viewed with a laser‐scanning confocal microscope.^3^ In brief, the paraffin‐embedded sections were incubated in PBS containing 10% goat serum for 1 hour and were consecutively incubated with primary antibody against RP105. Slices were maintained with secondary antibodies including FITC‐conjugated goat anti‐rabbit IgG (1:500, BA1105, Boster Biological Technology Co.Ltd.). DAPI was used to stain nucleus. Images were acquired using a laser‐scanning confocal microscope (Leica).

### 2.13 Luciferase reporter assay

To verify that RP105 was a direct target of miR‐383, the full‐length 3′‐UTR of RP105, which contains the predicted target binding sites either as wild‐type pmir‐RP105‐3′‐UTR or as a mutant (RP105 3′‐UTR‐MT), was cloned into pmirGLO luciferase vector (Promega).^27^, ^28^ Then, the wild‐type or mutant RP105 3′‐UTR and pRL‐SV40 Renilla luciferase control vector were co‐transfected in HEK293 cells with the miR‐383 mimics or the miRNA control for 48 hours. A luciferase reporter system (Promega) was performed to measure luciferase activity. The data were expressed as a percentage of the vector group.

### 2.14 Western blotting assay

Western blotting analysis was used to detect protein expression as previously described.^4^, ^14^ Myocardial samples were homogenized and lysed in RIPA lysis buffer (Beyotime Institute of Biotechnology) at a mass/volume ratio of 100 mg/mL. Protein was then extracted, and protein concentration was detected using a bicinchoninic acid (BCA) protein assay. Next, we used 10% SDS‐PAGE to separate proteins, and 50 µg of extracted proteins in each group were electrophoretically transferred onto PVDF membrane (EMD Millipore). Afterwards, the membranes were blocked by 5% skimmed milk in TBST for 2 hours at 37°C. Thereafter, the membranes were incubated with antibodies against RP105, p‐PI3K, t‐PI3K, p‐AKT, t‐AKT, NLRP3, cleaved caspase‐1, pro‐caspase‐1, cleaved IL‐1β, pro‐IL‐1β, IL‐18 and GAPDH. The horseradish peroxidase‐conjugated secondary antibody was added for another 2 hours at 37°C. The protein bands were visualized using an enhanced chemiluminescence (ECL) system (Thermo Fisher Scientific, Inc.).

### 2.15 Quantitative Real‐time PCR

Quantitative Real‐time PCR (qRT‐PCR) was used to detect mRNA expressions.^3^, ^4^, ^14^ In detail, total RNA was extracted from heart samples using TRIzol^®^ Reagent (Invitrogen) after indicated treatments. qRT‐PCR was manipulated by using a SYBR Green Master Mix Kit (cat. No. 4472918; Thermo Fisher) on a 7500 ABI Prism system. The PCR conditions were listed as follows: initial denaturing at 50°C for 2 minutes, 40 cycles of denaturation at 95°C for 30 seconds and final extension at 60°C for 30 seconds. The mRNA expressions of miR‐383 and RP105 were normalized to U6 and β‐actin, respectively. The primers were listed as follows:

miR‐383 Loop‐Primer:

5′‐GTCGTATCCAGTGCAGGGTCCGAGGTATTCGCACTGGATACGACTTACAGTC‐3′

Forward: 5′‐ TGCGCCAGATCAGAAGGTGAC‐3′;

RP105‐Forward: 5′‐AGCTTTAATGCCTTGCCTACG‐3′

Reverse: 5′‐ATGCTTCAGTGCCTTAGGCC‐3′;

β‐actin‐Forward 5′‐CACGATGGAGGGGCCGGACTCATC‐3′

Reverse, 5′‐TAAAGACCTCTATGCCAACACAGT‐3′

U6‐Forward, 5′‐CAAATTCGTGAAGCGTT‐3′

Reverse, 5′‐TGG TGTCGTGGAGTCG‐3′.

### 2.16 Statistical analysis

SPSS 17.0 was used for statistical analysis.^3^, ^26^ Data were expressed as means ± SD. Student's *t* test was used for between‐group comparisons. The statistical comparisons among multiple groups were performed with analysis of variance (ANOVA) followed by a post hoc Tukey's test. *P* value <.05 was considered statistically significant.

## 3 RESULTS

### 3.1 PIP attenuated cardiac injury induced by I/R insult

**Figure 1:**
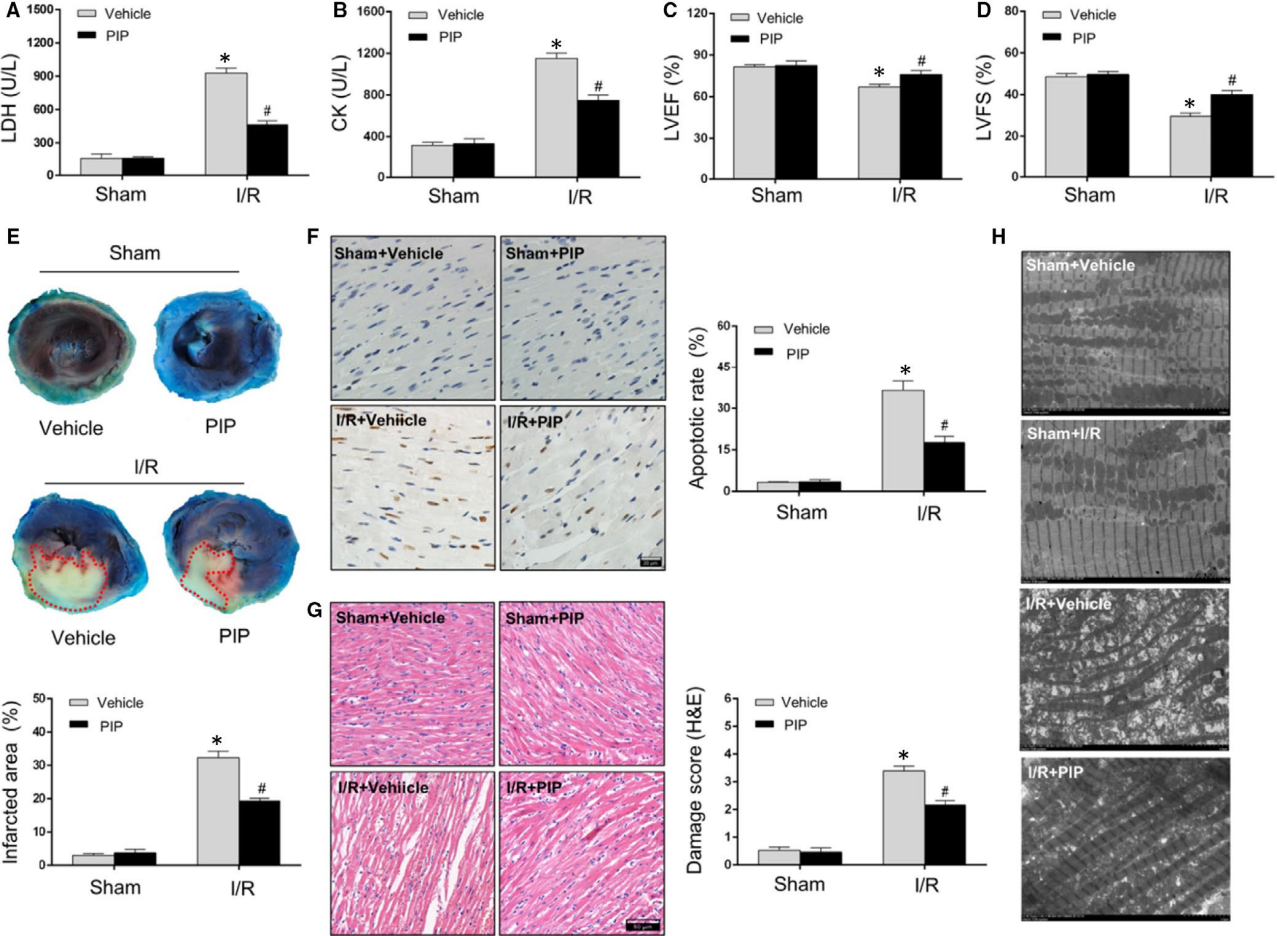
Piperine (PIP) attenuated cardiac injury induced by I/R insult. A and B, Myocardium death was estimated by Lactate dehydrogenase (LDH) and creatine kinase (CK) releases after PIP treatment with or without myocardial ischaemia/reperfusion (I/R) injury (MIRI) (n = 6). C and D, Echocardiographic results of LV ejection fraction (LVEF) and factional shortening (LVFS) indicating cardiac function after PIP treatment in response to MIRI or sham operation. E, Representative section images (upper panel) and averaged data of the ratio of infarction area to total left ventricular area (lower panel) detected by Evans blue plus TTC staining (n = 4). Evans blue‐stained areas (dark blue) presented non‐infarcted area; TTC‐stained regions (pale white) were infarcted tissue. F, Representative photomicrographs (left panel) and averaged data of the percentage of TUNEL‐positive cells (right panel) in rat heart with or without PIP subjected to I/R injury (n = 5). TUNEL staining (brown) indicates apoptotic nuclei; DAPI counterstaining (blue) indicates total nuclei. 400× magnification. Scale bar = 20 μm (n = 5). G, Representative pathological pictures (left panel) by H&E staining and H&E damages scores (right panel) in rat heart with or without PIP subjected to sham or I/R injury (n = 5). 200× magnification. Scale bar = 50 μm. H, Representative mitochondrial morphologies as detected by TEM either subjected to I/R injury or not (n = 4). Scale bar = 5 μm. The data are expressed as the mean ± SD. *P < .05, compared with the sham group; ^#^ *P* < .05, compared with the I/R + vehicle group

To evaluate the possible effects of PIP on myocardial I/R injury, rat heart with or without PIP pre‐treatment were subjected to 30 minutes of ischaemia followed by 4 hours of reperfusion or sham operation. The releases of LDH/CK, as the common markers of myocardial damage after MIRI, were detected to assess cardiomyocytes death.^3^ As shown in Figure 1A,B, the great releases of LDH and CK caused by I/R injury was limited after PIP treatment. Then, for the measurement of cardiac function, echo assay was performed to evaluate LVEF (Figure 1C) and LVFS (Figure 1D) in I/R‐ or sham‐suffered rat heart with or without PIP administration. In MIRI condition, LVEF and LVFS were markedly decreased relative to the sham groups, both of which however were partly reversed after PIP treatment (Figure 1C,D). Moreover, Evans blue plus TTC staining was used to evaluate myocardial infarction after MIRI.^3^ As displayed in Figure 1E, myocardial infarcted areas were strengthened after I/R, which was repressed by PIP administration. Thereafter, apoptosis was detected by TUNEL staining.^26^ The results from Figure 1F demonstrated that the minimal TUNEL‐positive cells and quantitative apoptotic rate were observed in the sham group, which was further aggravated in MIRI. PIP treatment, however, dampened apoptosis in comparison with that myocardium was stimulated with vehicle under I/R. Furthermore, the alternations of myocardial inflammation and morphology demonstrated by H&E staining were performed to measure MIRI. As exhibited in Figure 1G, in the sham group, morphologic changes of heart tissue were completely maintained, in which myocardial fibres were intact, and were arranged regularly with no apparent epimorphosis, necrosis or inflammatory infiltrations. However, after I/R suffering, we observed that myocardial fibres were partially disturbed and were disorganized in parallel with interstitial oedema, neutrophil infiltration, cell fell apart and myocyte necrosis. Interestingly, these pathological damages after MIRI were prominently ameliorated in the presence of PIP pre‐treatment (Figure 1G, left panel). Moreover, the damage scores from H&E staining in the PIP‐treated rat heart were also lower, when compared with the vehicle + I/R group (Figure 1G, right panel). Next, mitochondrial structure visualized by electron microscopy was carried out to indicate myocytes damages in MIRI (Figure 1H). The result demonstrated that I/R markedly resulted in mitochondrial swelling, vacuoles and rupture of mitochondria, all of which were partially rescued in the PIP‐treated myocardium relative to the I/R + vehicle group (Figure 1H). Collectively, abovementioned results indicated that PIP pre‐treatment was able to increase cardiomyocytes survival and repress myocardial dysfunction and damage under I/R injury.

### 3.2 PIP treatment alleviated pyroptosis during I/R injury

**Figure 2:**
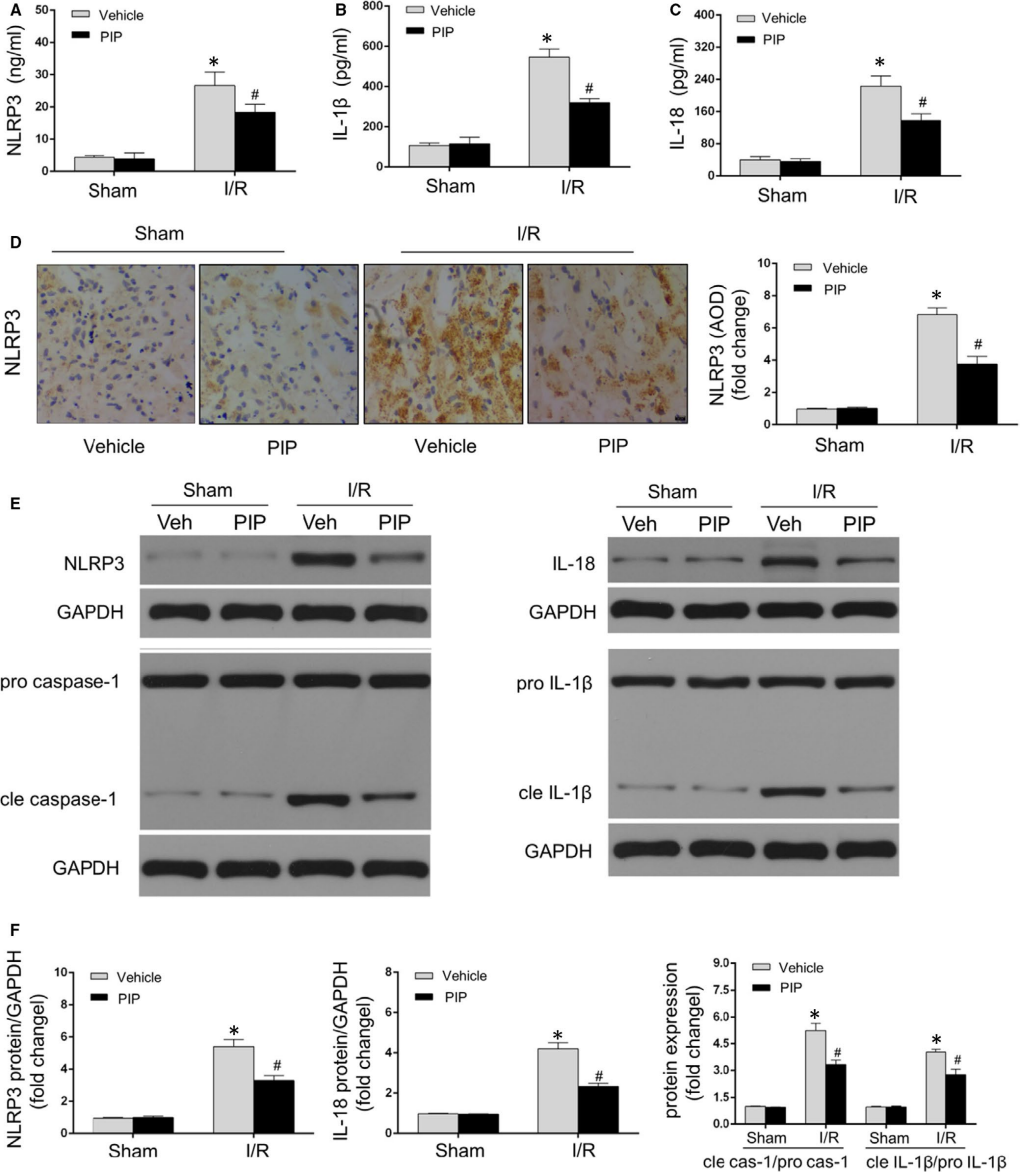
Piperine (PIP) treatment alleviated pyroptosis during I/R injury. A through C, The levels of pyroptosis‐related inflammatory mediators including NLRP3, IL‐1β and IL‐18 that detected by ELISA assay (n = 6). D, Immunohistochemistry assay (left panel) and quantitive data (right panel) illustrated NRLP3 expression after PIP administration under the sham or I/R operation (n = 4). 400× magnification. Scale bar = 20 μm. E and F, Representative Western blots (E, upper panel) and quantitative results (F, lower panel) of protein expressions of pyroptosis‐related mediators including NLRP3, cleaved/pro‐caspase‐1, cleaved/pro‐IL‐1β and IL‐18 that detected by Western blotting assay (n = 6). The data are expressed as the mean ± SD. *P < .05, compared with the sham group; ^#^ *P* < .05, compared with the I/R + vehicle group

A large range of studies have verified that I/R‐caused myocardial damage was tightly related to the activation of NLRP3/caspase‐1‐dependent pyroptosis.^7^, ^10^, ^12^ When NLRP3 is stimulated, inactive pro‐caspase‐1 is hydrolysed into activated caspase‐1, and acted as a crucial enzyme to stimulate IL‐1β and IL‐18, which thereby resulted in cell death in the initiation of MIRI.^7^, ^10^, ^12^ To scrutinize whether PIP have an influence on myocardial I/R injury in a pyroptosis‐related way, the levels of myocardial NLRP3, cleaved caspase‐1, cleaved IL‐1β and IL‐18 were assessed after I/R disorder. As shown in Figure 2A throughout C, the concentrations of pro‐inflammatory mediators of myocardial pyroptosis including NLRP3, IL‐1β and IL‐18 remained at higher level in the I/R group relative to the sham group. All of these augments determined by ELISA analysis were reduced on PIP‐treated rat heart in MIRI. In similar, immunohistochemistry analysis illustrated lower expression of NLRP3 after PIP treatment following MIRI relative to the vehicle + I/R group (Figure 2D). Accordingly, we further examined the proteins expressions of pyroptotic markers by Western blotting assay. As shown in Figure 2E,F, the proteins levels of NLRP3, cleaved (cle) caspase‐1, cle IL‐1β and IL‐18 were enhanced in response to MIRI relative to the sham group. Notably, I/R‐induced increases of those pyroptotic proteins were depressed after PIP treatment that subjected to I/R. Moreover, there exhibited no significant difference for pro‐caspase‐1 and pro‐IL‐1β expressions among the groups. Therefore, above date indicated that PIP pre‐treatment could mitigate myocardial pyroptosis induced by I/R injury.

### 3.3 PIP pre‐treatment repressed miR‐383 and activated RP105/PI3K/AKT pathway

**Figure 3:**
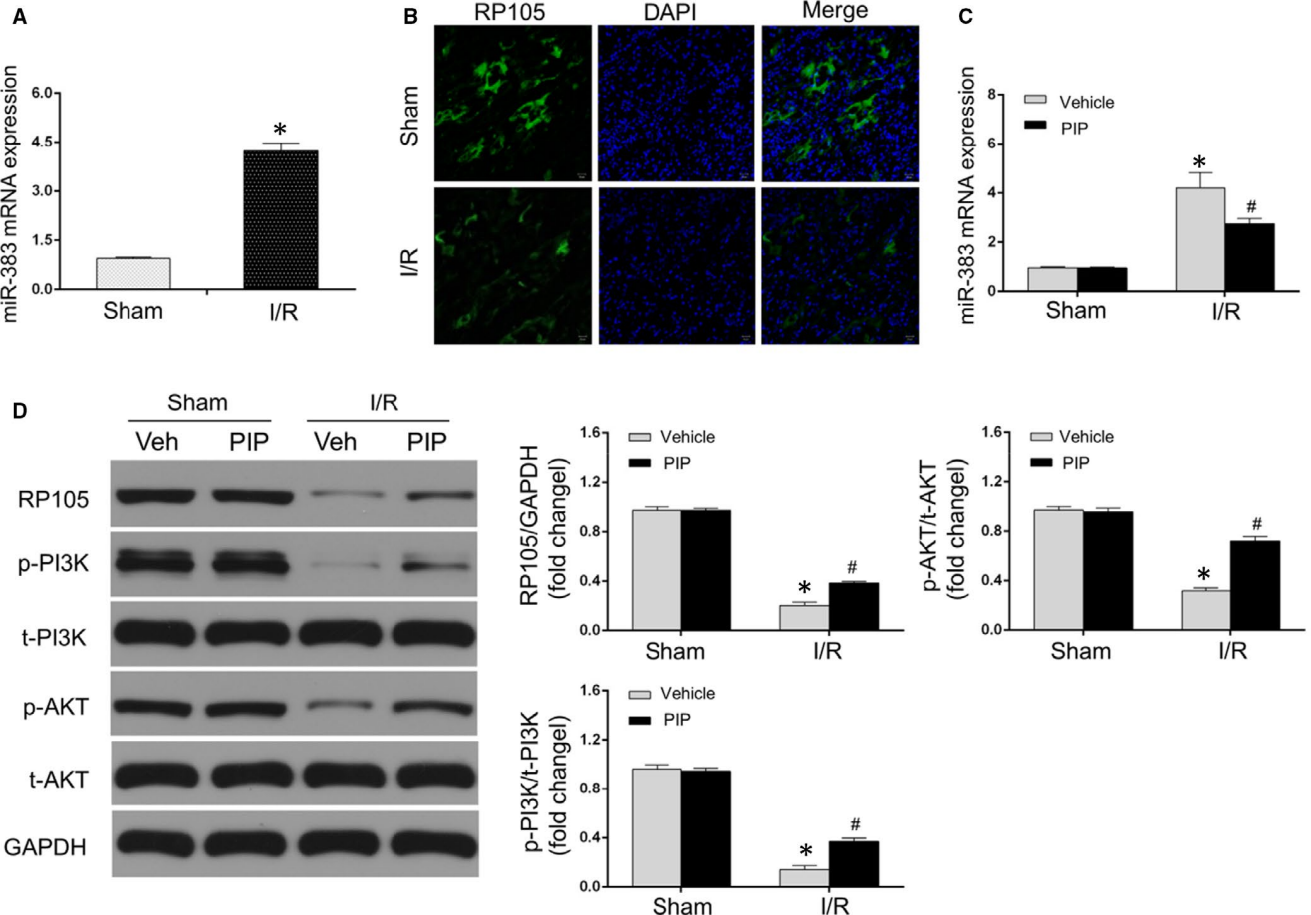
Piperine (PIP) repressed miR‐383 and activated RP105/PI3K/AKT signalling pathway. A, The mRNA level of miR‐383 in heart tissue at post‐I/R injury (n = 6; *P < .05 vs sham group). B, Respective representations of immunofluorescent images visualizing RP105 (green) and DAPI‐labelled nuclei of cardiomyocytes (blue) under I/R and sham conditions (400× magnification; scale bar = 20 μm) (n = 4). C, The mRNA level of miR‐383 in heart tissue after PIP treatment with or without I/R injury (n = 6). D, Representative Western blots (left panel) and quantitative results (right panel) of protein levels of RP105, p‐PI3K, t‐PI3K, p‐AKT, t‐AKT and GAPDH that detected by Western blotting assay (n = 6). The data are expressed as the mean ± SD. *P < .05, compared with the sham group; ^#^ *P* < .05, compared with the I/R + vehicle group

Previous studies demonstrated that PIP could exert multiple pharmacological effects through miRNA‐dependent manners.^16^, ^17^ Of note, recent investigation reported that miRNA‐mediated changes of RP105/PI3K/AKT pathway might participate in the progression of myocardial ischaemia.^25^ To further detect the possible molecular mechanisms of PIP in alleviating MIRI and pyroptosis, we first highlighted the expressions of miR‐383 and RP105/PI3K/AKT pathway. As shown in Figure 3A, the expression of miR‐383 at mRNA level was much higher in rat heart undergoing I/R injury when compared with that in the sham group. Moreover, according to immunofluorescent analysis, lower level of RP105 was visualized in the heart subjected to I/R insult relative to sham group (Figure 3B). These data suggested that the alterations of miR‐383 and RP105 seemed to play crucial contributions during MIRI.

Next, further research was designed to detect whether PIP regulated miR‐383 and RP105‐related pathways in MIRI. The results showed that PIP pre‐treatment at myocardial I/R condition repressed miR‐383 mRNA (Figure 3C) and increased RP05 protein (Figure 3D) compared with that in the I/R + vehicle group. The similar trends of protein expressions of PI3K/AKT pathways, which acted as a crucial downstream mediator of RP105, were exhibited after PIP treatment under I/R injury. As shown in Figure 3D, PIP greatly rescued I/R‐caused decreases of p‐PI3K and p‐AKT in I/R injury. Nevertheless, no significant difference for the levels of t‐PI3K and t‐AKT was observed between different groups. Thus, these data demonstrated that PIP could repress miR‐383 and activate RP105/PI3K/AKT pathway in MIRI. Therefore, we further speculated that I/R‐induced myocardial pyroptosis and death were capable to be ameliorated after PIP treatment, possibly through the miR‐383 and RP105/PI3K/AKT pathway.

### 3.4 miR‐383 affected the RP105/PI3K/AKT pathway during pyroptosis under MIRI

**Figure 4:**
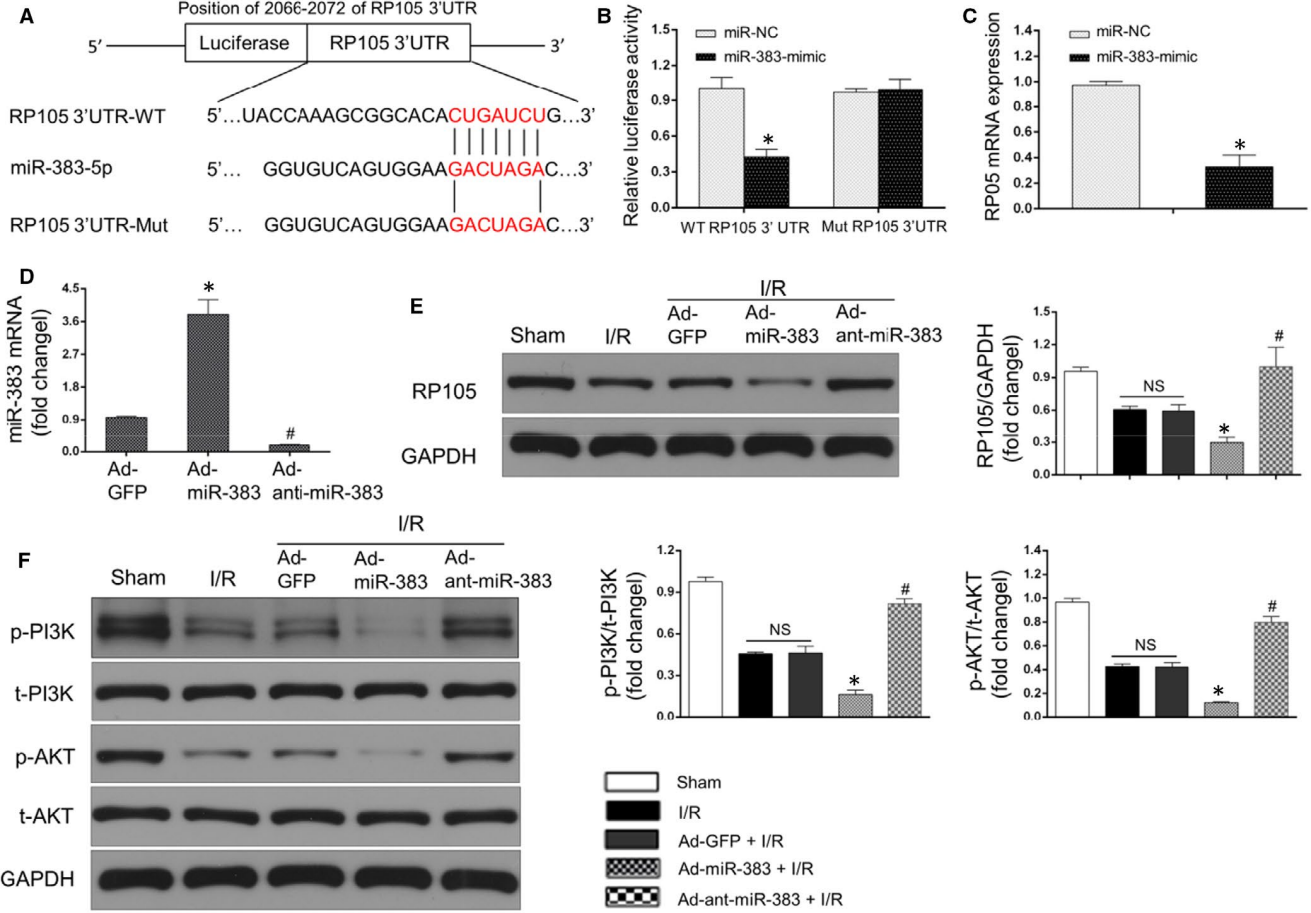
miR‐383 affected RP105/PI3K/AKT pathway under myocardial ischaemia/reperfusion (I/R) injury (MIRI). A, miR‐383 contains a site complementary of RP105, and the sequences of wild‐type and mutant 3′UTR of *RP105* was exhibited. B, Luciferase activity of RP105‐Wt and RP105‐Mut in HEK293 cells after miR‐383 transfection (n = 6, *P < .05). C, The mRNA level of RP105 measured by qRT‐PCR in cardiomyocytes (n = 6, *P < .05, compared with the miR‐NC group). D, The mRNA level of miR‐383 in heart in the presence of Ad‐GFP or Ad‐miR‐383 or Ad‐anti‐miR‐383 transfection (n = 6, *^#^ *P* < .05, compared with the Ad‐GFP group). E, Western blots showing RP105 protein expression after miR‐383 knockdown or miR‐383 overexpression in MIRI (n = 6, *^#^ *P* < .05, compared with I/R + Ad‐GFP group; NS means no significance). Left: representative bots; right: quantitative analysis. F, Western blots showing the protein levels of p‐PI3K, t‐PI3K, t‐AKT and p‐AKT after miR‐383 knockdown or miR‐383 overexpression in MIRI (n = 6, *^#^ *P* < .05, compared with I/R + Ad‐GFP group; NS means no significance)

Above studies have proved that the alterations of miR‐383 and RP105/PI3K/AKT signalling pathway might play crucial contributions during MIRI. However, more studies were still urged to confirm whether miR‐383 could affect myocardial pyroptosis and cardiomyocytes damage via the RP105/PI3K/AKT‐dependent pathway under MIRI disorder. By utilizing TargetScan and bioinformatics analysis, we found a binding site of miR‐383 seed region in RP105 mRNA (Figure 4A). Then, luciferase activity assay was performed to validate RP105 as a target gene of miR‐383. The data suggested that luciferase activity in wild‐type (WT) vector group was repressed after miR‐383 mimics transfection, which was not altered in mutant vector compared with the scramble group (Figure 4B). In addition, RP105 mRNA level was suppressed in cardiomyocytes after miR‐383 mimic transfection relative to that in the transfection of miRNA scramble (Figure 4C). These data indicated that RP105 was a target gene of miR‐383.

Our prior study reported that the activation of RP105/PI3K/AKT pathway might play protective effects in MIRI.^24^ In addition, miRNA‐mediated change of RP105/PI3K/AKT was verified to be involved in ischaemia.^25^ However, it was not clear if miR‐383‐mediated changes of RP105/PI3K/AKT axis participated in MIRI and pyroptosis. Firstly, to explore the impact of miR‐383 on RP105/PI3K/AKT pathways, we performed controlled gain‐ and loss‐of function experiments to depicture the roles of miR‐383 in MIRI. As shown in Figure 4D, rat hearts were transfected with either Ad‐miR‐383 or Ad‐anti‐miR‐383 to up‐regulate or down‐regulate miR‐383, respectively. Then, the protein expression of RP105 was observed after controlled miR‐383 levels. The results from Western blotting assay revealed that miR‐383 overexpression diminished and miR‐383 knockdown promoted RP105 protein under MIRI compared with the Ad‐GFP + I/R group (Figure 4E). Similarly, much less protein expressions of p‐PI3K and p‐AKT were observed in rat hearts on the Ad‐miR‐383 plus I/R group, while it was distinctly reversed when the heart was infected with Ad‐anti‐miR‐383, in comparison with the Ad‐GFP + I/R group (Figure 4F). These findings thus revealed that miR‐383 directly interacted with RP105 and causally inhibited RP105‐mediated PI3K/AKT signalling pathways.

**Figure 5:**
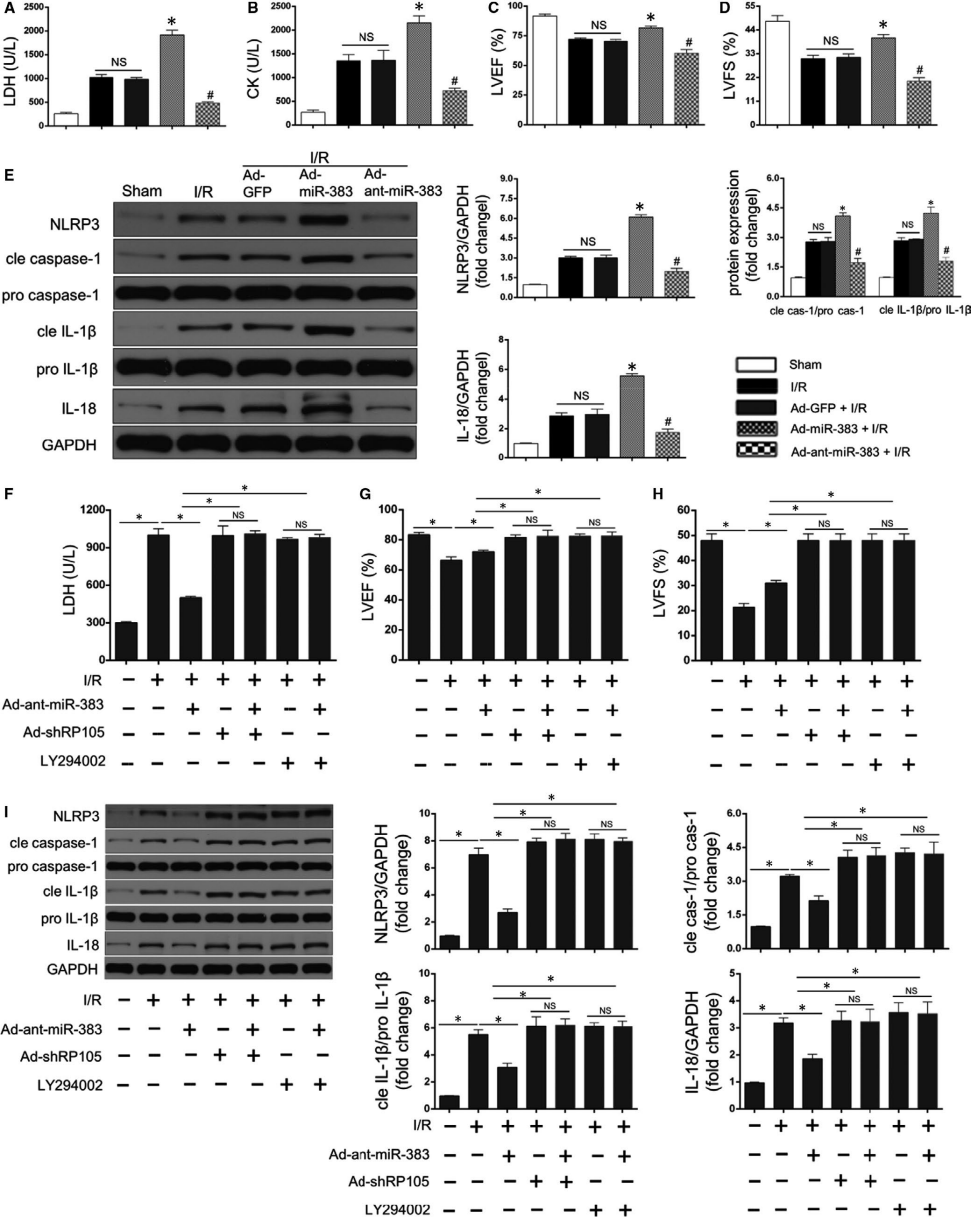
miR‐383 regulated pyroptosis through RP105/PI3K/AKT‐dependent pathway under myocardial ischaemia/reperfusion (I/R) injury (MIRI). A and B, Myocardium death estimated by Lactate dehydrogenase (LDH)/creatine kinase (CK) after miR‐383 overexpression or miR‐383 inhibition in response to MIRI (n = 6, *^#^ *P* < .05, compared with I/R + Ad‐GFP group; NS means no significance). C and D, Echocardiographic results of LV ejection fraction (LVEF) and factional shortening (LVFS) indicating cardiac function after controlled miR‐383 levels in MIRI (n = 6, *^#^ *P* < .05, compared with I/R + Ad‐GFP group; NS means no significance). E, Western blots showing the proteins expressions of NLRP3, cleaved caspase‐1/pro‐caspase‐1, cleaved IL‐1β/pro‐IL‐1β and IL‐18 after miR‐383 silencing or miR‐383 overexpression under MIRI condition (n = 6, *^#^ *P* < .05, compared with I/R + Ad‐GFP group; NS means no significance). Left: representative bots; right: quantitative analysis. F, Myocardium death was estimated by LDH with or without Ad‐anti‐miR‐383 transfection in the presence of RP105 silencing by AdshRP105 transfection or PI3K/AKT inhibition by LY294002 in MIRI (n = 6, *P < .05, compared with corresponding controls; NS means no significance). G and H, Echocardiographic results of LVEF and LVFS indicated cardiac function with or without miR‐383 knockdown in the presence of RP105 silencing or PI3K/AKT inhibition in MIRI (n = 6, *P < .05, compared with the corresponding controls; NS means no significance). I, The protein levels of pyroptosis‐related mediators with or without Ad‐anti‐miR‐383 transfection in the presence of RP105 silencing or PI3K/AKT inhibition under MIRI (n = 6, *P < .05, compared with the corresponding controls; NS means no significance)

Additionally, more experiments were carried out to better understand potential biological functions of miR‐383/RP105/AKT pathway in MIRI and myocardial pyroptosis. As revealed in Figure 5A throughout D, the hallmarks for cardiac death and dysfunctions, as exhibited by LDH/CK release (Figure 5A,B) and LVEF/LVFS (Figure 5C,D), respectively, were greatly exacerbated after MIRI. Interestingly, these parameters were reinforced after Ad‐miR‐383 transfection but were limited after Ad‐anti‐miR‐383 treatment compared with Ad‐GFP + I/R group under MIRI (Figure 5A throughout D). Furthermore, the influences of miR‐383 on myocardial pyroptosis in response to MIRI were also highlighted. Interestingly, a similar trend of pyroptosis‐associated mediators was demonstrated in Figure 5E. In details, relative to the Ad‐GFP + I/R group, the protein expressions of NLRP3, cle caspase‐1, cle IL‐1β and IL‐18 were further facilitated after miR‐383 overexpression and were limited after miR‐383 knockdown. Finally, we detect whether miR‐383 regulated myocardial pyroptosis and I/R injury via the RP105/PI3K/AKT‐related way. The results showed that RP105 knockdown by AdshRP105 or PI3K/AKT inhibition abolished cardiac‐protective and pyroptosis‐reduced functions resulting from miR‐383 knockdown. As exhibited in Figure 5F throughout H, miR‐383 inhibition‐induced alleviations of LDH/CK and LVEF/LVFS were reverted in AdshRP105‐ or LY294002‐treated hearts compared with the respective controls. Importantly, RP105 knockdown or PI3K/AKT inhibition also reversed the decreases of myocardial pyroptotic mediators in MIRI (Figure 5I). Therefore, these data validated that miR‐383 regulated pyroptosis and MIRI mainly through the RP105/PI3K/AKT‐dependent pathway.

### 3.5 miR‐383 increase or RP105 inhibition abolished the protective effects of PIP on myocardial pyroptosis and I/R injury

**Figure 6:**
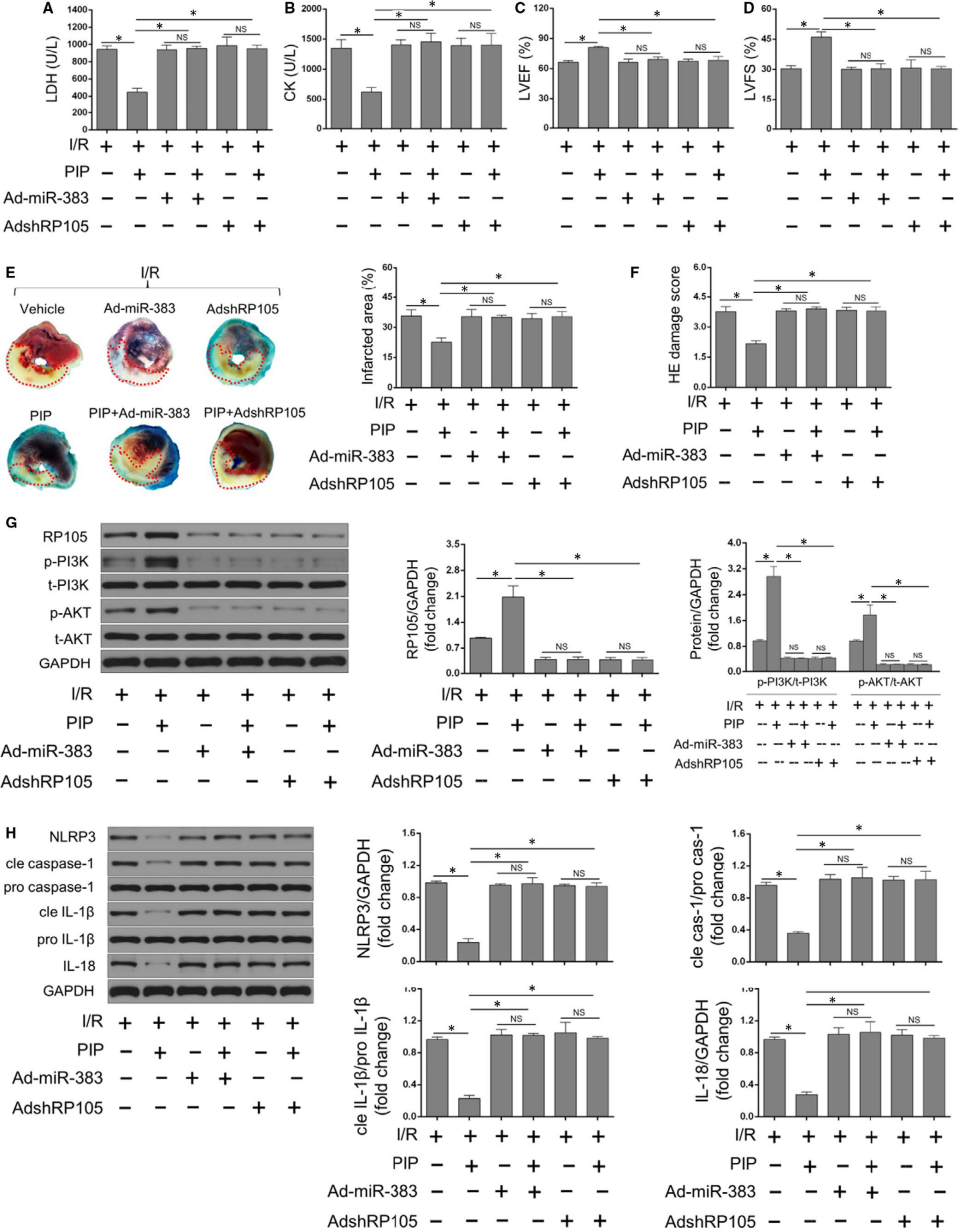
miR‐383 increase or RP105 inhibition abolished the protective effects of Piperine (PIP) on myocardial pyroptosis and I/R injury. Lactate dehydrogenase (LDH)/creatine kinase (CK) concentration (A and B) and cardiac function (C and D) after PIP or vehicle treatment followed by miR‐383 increase or RP105 silencing in response to myocardial ischaemia/reperfusion (I/R) injury (MIRI) (n = 6, *P < .05, compared with the corresponding controls; NS means no significance). E, Myocardial infarcted areas detected by Evans blue + TTC staining after PIP or vehicle treatment in the presence of miR‐383 increase or RP105 silencing in MIRI (n = 4, *P < .05, compared with the corresponding controls; NS means no significance). F, H&E damages scores in rat heart with or without PIP treatment in the presence of miR‐383 increase or RP105 silencing in MIRI (n = 4, *P < .05, compared with the corresponding controls; NS means no significance). G, The protein levels of RP105/PI3K/AKT signalling pathways with or without PIP treatment in the presence of miR‐383 increase or RP105 silencing subjected to MIRI (n = 6, *P < .05, compared with the corresponding controls; NS means no significance). H, The protein levels of pyroptosis‐associated mediators with or without PIP treatment in the presence of miR‐383 increase or RP105 silencing after MIRI (n = 6, *P < .05, compared with the corresponding controls; NS means no significance)

Finally, further experiments were performed to confirm whether the miR‐383/RP105/AKT pathway served as causative molecular mechanisms for PIP‐drove repression of MIRI and pyroptosis. I/R‐suffered rat hearts were pre‐treated with Ad‐miR‐383 or AdshRP105 with or without PIP, respectively. According to the results from LDH/CK releases, echo determination, Evans blue + TTC assay and H&E damage scores, respectively, we observed that PIP treatment dramatically attenuated myocardial deaths (Figure 6A,B), cardiac dysfunction (Figure 6C,D), infarction areas (Figure 6E) and pathological abnormalities (Figure 6F). More importantly, these protective effects of PIP were abolished in the presence of miR‐383 overexpression or RP105 inhibition under MIRI. On the other hand, similar results were also determined for the RP105/PI3K/AKT signalling pathways and myocardial pyroptosis. As revealed in Figure 6G,H, PIP‐elicited activations of RP105/PI3K/AKT pathway (Figure 6G) and PIP‐induced repressions of pyroptotic markers (Figure 6H) were abrogated after miR‐383 increase or RP105 repression under MIRI. Collectively, these findings thoroughly indicate that PIP pre‐treatment alleviates pyroptosis and MIRI mainly through the miR‐383/RP105/AKT‐dependent pathway.

## 4 DISCUSSION

Ample data have suggested that PIP has pleiotropic impacts in multiple cardiovascular diseases.^13^, ^14^ In a rat model of isoproterenol (ISO)‐induced myocardial infarction, Dhivya and his colleagues demonstrated that PIP acted as a potent therapeutic agent with its antioxidant action.^31^ Ma et al,^14^ indicated that PIP treatment attenuated pathological cardiac fibrosis after pressure overload. Moreover, PIP pre‐treatment was reported to reduce oxidative stress, inflammation and apoptosis in DOX‐induced cardiotoxicity.^13^ Interestingly, the latest study has indicated that PIP exerts pivotal effects on the pathological process of I/R as well.^20^, ^21^ For instance, in a rat model of cerebral I/R injury, Zou et al^32^ uncovered that PIP pre‐treatment protected neurocytes from I/R damages by regulating complement and coagulation cascades. Vaibhav et al^33^ found that PIP administration was able to salvage ischaemic penumbral zone neurons by virtue of its anti‐inflammatory property. However, it has not been investigated whether PIP participated in the development of MIRI, and potential pathogenetic mechanisms were still unknown. Recently, some evidences demonstrated that PIP was an important mediator for the initiation of pyroptosis. Liang et al^19^ demonstrated that PIP could protect macrophages from pyroptosis and reduce IL‐1β release, thereby becoming a potential therapeutic agent against bacteria sepsis. In terms of the possible roles of PIP in I/R challenge and pyroptosis, we thus hypothesized that PIP could play protections and inhibit pyroptosis in response to I/R. Consequently, our present experiments clearly provided evidences that PIP treatment has advantageous effects against I/R‐caused pyroptosis and thus ameliorates MIRI.

Timely and effectively reperfusion acts as most efficacious remedy for AMI.^4^, ^6^ However, accompanying MIRI largely offset this beneficial effect.^4^, ^6^ Currently, findings ways to mitigate the influences of reperfusion injury were still needed.^5^, ^9^ As well investigated, programmed cell death exerted essential contributions in the process of MIRI.^7^, ^27^ Three distinct forms of programmed cell death have been indicated, namely necrosis, apoptosis and autophagy.^6^, ^7^, ^8^, ^34^ Necrosis, previously verified as a un‐regulatory avenue of cell death, has been seen as a tightly modulated process and obtained great attentions in MIRI.^34^, ^35^ Programmed necrosis mainly contains necroptosis, mitochondrial permeability transition‐associated necrosis, ferroptosis and pyroptosis.^7^, ^35^, ^36^ Currently, myocardial pyroptosis is considered to be one of the pivotal pathogenetic mechanisms of MIRI and has drawn considerable attentions during MIRI.^6^, ^7^, ^10^, ^11^, ^36^ Pyroptosis acts as a pro‐inflammatory cell death and was characterized as NLRP3‐caspase‐1‐dependent response.^6^, ^7^, ^10^, ^11^, ^36^ NLR family pyrin domain containing 3 (NLRP3) inflammasome is a macromolecular complex that composed of NLRP3, an adaptor protein apoptosis‐associated speck‐like protein (ACS) and caspase‐1.^6^, ^7^, ^10^, ^11^, ^36^ Currently, four inflammasomes have been indicated including NLRP1, NLRP3, NLRP4 and AIM2, among which NLRP3 inflammasome has been widely identified, and was closely associated with the development of MIRI.^6^, ^7^, ^10^, ^11^, ^36^ When NLRP3 is activated in MIRI, it interacted with ACS and recruited pro‐caspase‐1 to assemble an inflammasome.^6^, ^7^, ^10^, ^11^, ^36^ Subsequently, inactive pro‐caspase‐1 is hydrolysed into activated caspase‐1. Activated caspase‐1 acted as a crucial enzyme to stimulate specific targets including IL‐1β and IL‐18 and recruits pro‐inflammatory immune cells and expanded pro‐inflammatory responses, thereby leading to cell death in the initiation of MIRI.^6^, ^7^, ^10^, ^11^, ^36^ Increasing evidence suggested that the action of pyroptosis was closely associated with myocardial death, poor ventricular remodelling and dysfunction after MIRI.^6^, ^7^ Notably, present study showed that MIRI dramatically induced myocardial damage and promoted levels of pyroptosis‐related mediators. However, PIP treatment reduced the protein expressions of pro‐pyroptotic mediators like NLRP3, cleaved caspase‐1, cleaved IL‐1β and IL‐18, and ameliorated the parameters in association with the severity of MIRI. Thus, PIP has the ability to protect cardiomyocytes from I/R injury mainly by mitigating I/R‐induced pyroptosis.

Based on the above findings, we further explored the potential molecular mechanisms by which PIP exerted its protective effects against I/R injury and pyroptosis. miRNAs are a group of non‐coding RNAs and could repress target genes via direct bonding to 3′UTR of mRNA.^10^, ^27^, ^28^ Previous studies indicated that PIP exerted pharmacological effects via miRNA‐dependent manners.^16^, ^17^ However, the exacted effects of PIP via the miRNA‐related ways in MIRI are not clear. Interestingly, an altered expression of miRNA, miR‐383, was identified in the I/R‐treated rat heart in our present study. After myocardial I/R insult, the level of miR‐383 was markedly increased. miR‐383 overexpression facilitated and miR‐383 inhibition alleviated myocardial death and pyroptosis, suggesting an important effect of miR‐383 in MIRI. Furthermore, PIP administration reduced miR‐383 expression followed with the attenuation of cell death and pyroptosis. The rescued experiments further verified that the cardio‐protective roles of PIP against pyroptosis and MIRI were largely dependent on miR‐383‐mediated signalling pathway. Therefore, this is the first study describing that PIP decreases pyroptosis and I/R‐induced cell damage in a miR‐383‐dependent approach.

As well known, PI3K and its downstream target serine/threonine kinase AKT belong to a conserved family of signal transduction enzymes.^3^, ^24^ Our prior study and other groups have proved that transmembrane protein RP105‐mediated activation of PI3K/AKT pathway was considered as important endogenous mechanisms in facilitating cell survival in myocardial ischaemia.^3^, ^24^, ^25^ Interestingly, the modulation of RP105/PI3K/AKT pathway arising from miRNA alternation in ischaemic insult has begun to be emerged.^25^ In consistent with previous explorations, we reported for the first time that the activation of RP105/PI3K/AKT pathway resulting from PIP‐initiated miR‐383 inhibition served as an important pro‐survival avenue against pyroptosis in MIRI. Our present study validated that pre‐treatment with PIP not only inhibited miR‐383 mRNA, but also up‐regulated the expressions of RP105, p‐PI3K and p‐AKT. miR‐383 overexpression repressed and miR‐383 silencing enhanced the RP105/PI3K/AKT pathway. Then, luciferase assays validated that RP105 was a direct target of miR‐383. Next, we observed that the protective roles of miR‐383 inhibition against I/R injury and pyroptosis were partly blocked in the presence of RP105 silence or PI3K/AKT inhibition, which suggested that miR‐383 regulated MIRI and pyroptosis via RP105/PI3K/AKT‐dependent method. Finally, our studies provided data that the cardio‐protective and anti‐pyroptotic efficacies of PIP were largely abolished under the conditions of miR‐383 increase or RP105/AKT inhibition in MIRI. Therefore, the above data indicated that PIP treatment alleviates pyroptosis and MIRI mainly through the miR‐383/RP105/AKT‐dependent approach. Notably, more studies are still urgent to verify whether other molecular mechanisms were implicated in PIP‐rendered protections against MIRI, and it may provide unrecognized insights in the development of MIRI.

In conclusion, our present study demonstrated that PIP pre‐treatment could ameliorate MIRI and attenuate pyroptosis in a miR‐383/RP105/AKT‐dependent approach (Supplementary material, Figure S3). In subsequent study, we will seek to clarify whether there are any other pathways or pathogenesis involved in the cardio‐protections of PIP in MIRI. Taken together, our present investigations indicate that PIP may be a prospective therapeutic option for the treatment of MIRI.

## CONFLICT OF INTEREST

The authors declare no conflicts of interest.

## AUTHOR CONTRIBUTIONS

**Xin Guo:** Conceptualization (equal); Data curation (equal); Formal analysis (equal); Funding acquisition (equal); Investigation (equal); Methodology (equal); Project administration (equal); Resources (equal); Software (equal); Supervision (equal); Validation (equal); Visualization (equal); Writing‐original draft (equal); Writing‐review & editing (equal). **Shan Hu:** Conceptualization (equal); Data curation (equal); Formal analysis (equal); Funding acquisition (equal); Investigation (equal); Methodology (equal); Project administration (equal). **Liu Ji Jun:** Conceptualization (supporting); Data curation (supporting); Formal analysis (supporting). **Ling Huang:** Resources (equal); Software (equal); Supervision (equal); Validation (equal); Visualization (equal); Writing‐original draft (equal). **Peng Zhong:** Conceptualization (supporting); Data curation (supporting); Formal analysis (supporting); Funding acquisition (supporting); Methodology (equal). **Zhi Xing Fan:** Resources (supporting); Software (supporting); Supervision (supporting); Validation (supporting); Visualization (supporting). **Ping Ye:** Investigation (equal); Methodology (equal); Resources (supporting); Software (supporting); Supervision (supporting); Validation (supporting). **Man Hua Chen:** Conceptualization (lead); Data curation (lead); Formal analysis (lead); Funding acquisition (lead); Investigation (lead); Methodology (lead); Project administration (lead).

## Supporting information

Figure S1‐S3

## Data Availability

Thedata will be made available after beenrequired upon request from the corresponding author.
