# Supplementary material for: Piperine protects against pyroptosis in myocardial ischaemia/reperfusion injury by regulating the miR‐383/RP105/AKT signalling pathway
Source: J Cell Mol Med. 2020 Nov 21;25(1):244–58. doi: 10.1111/jcmm.15953 (PMC7810957; doi:10.1111/jcmm.15953)
Supplement: Supplementary file 1 — Figure S1‐S3 [file JCMM-25-244-s001.docx]

**SUPPLEMENTARY MATERIAL**

SUPPLEMENTARY FIGURES

**Figure S1. Pre-treatment with PIP alleviated I/R-induced cardiac injury**


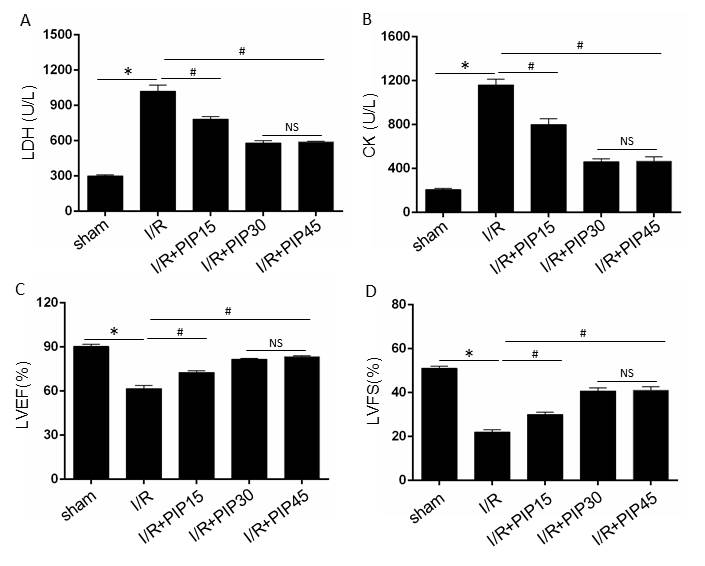


Figure S1. Pre-treatment with PIP alleviated I/R-induced cardiac injury. Serum levels of LDH (A) and CK (B) after PIP treatment (15, 30, and 45 mg/kg) under MIRI (n = 8). The effects of PIP treatment (15, 30, and 45 mg/kg) on cardiac LVEF (C) and LVFS (D) after I/R insult (n = 5). Data are expressed as the mean ± SD. *P<0.05, compared with the sham group; ^#^P<0.05, compared with the I/R. NS means no significant difference.

**Figure S2. Schematic representation of experimental protocol.**


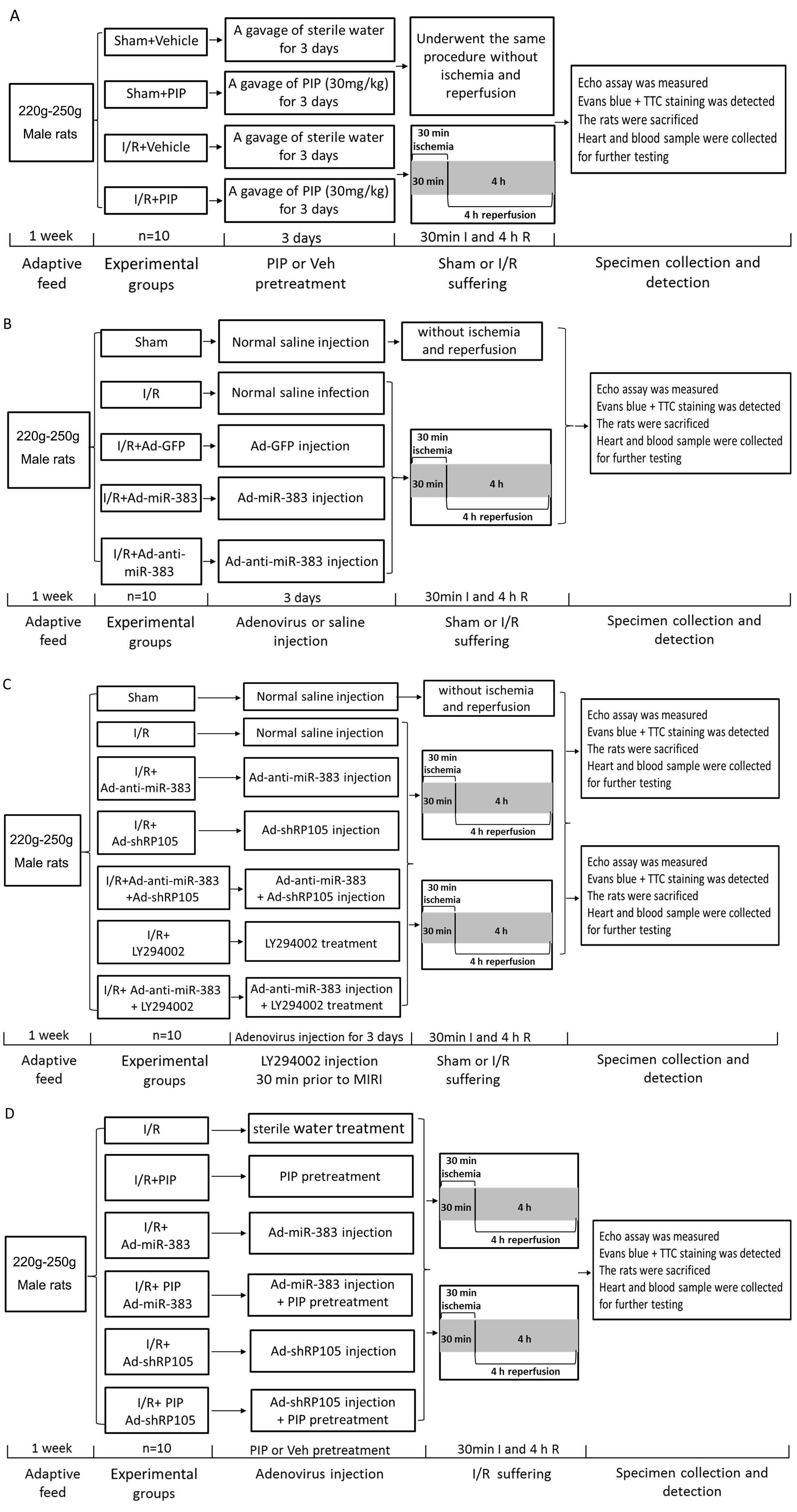


Figure S2. Schematic representation of experimental protocol. Diagram illustrates the groups of experimental animals, ischemia/reperfusion period, pretreatments and specimen collection time points.

**Figure S3. The proposed model of the effect of PIP on MIRI.**


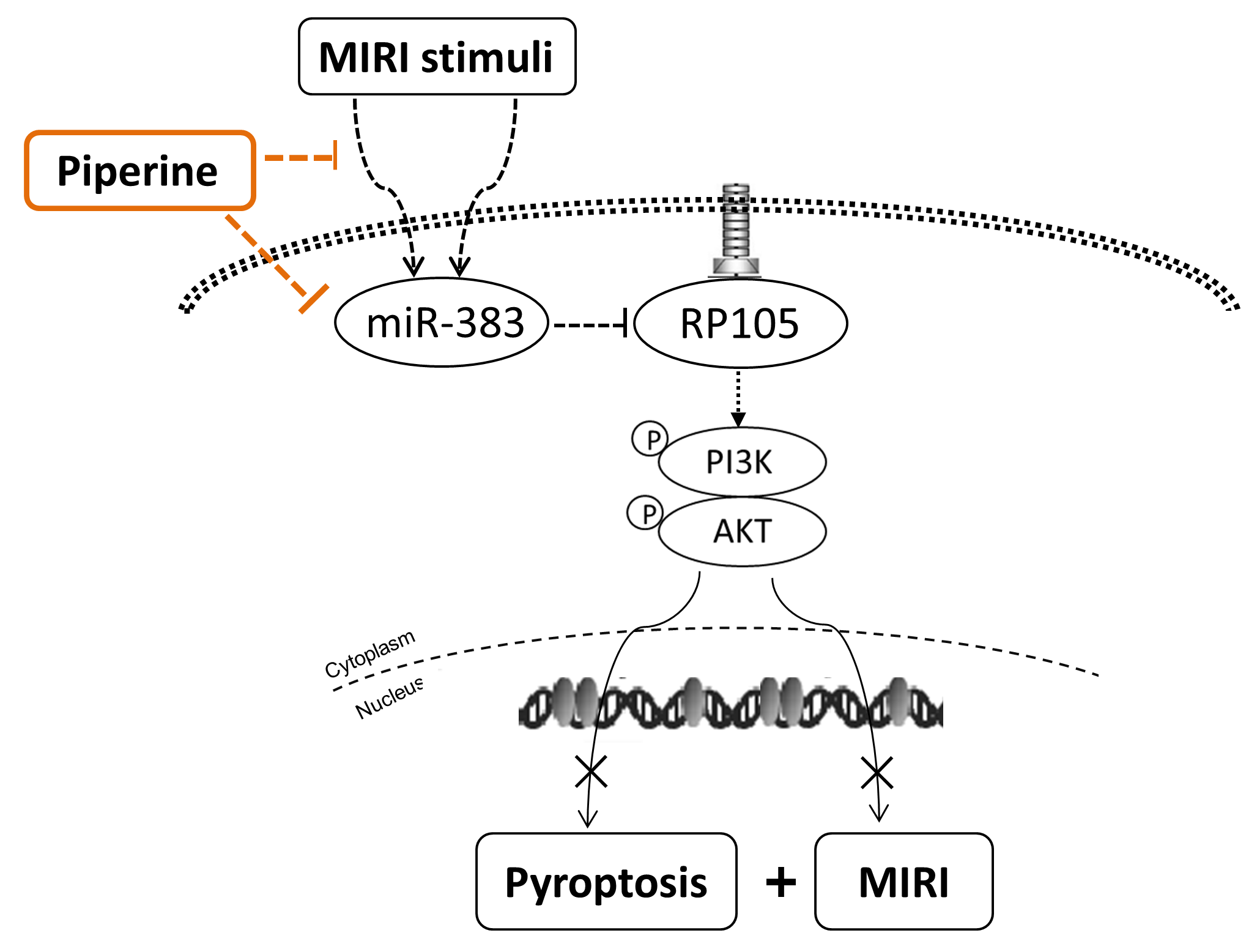


Figure S3. Representative proposed model of the effect of PIP on MIRI.
